# G6PDi-1 is a Potent Inhibitor of G6PDH and of Pentose Phosphate pathway-dependent Metabolic Processes in Cultured Primary Astrocytes

**DOI:** 10.1007/s11064-023-03964-2

**Published:** 2023-07-02

**Authors:** Patrick Watermann, Christian Arend, Ralf Dringen

**Affiliations:** 1 Centre for Biomolecular Interactions Bremen, Faculty 2 (Biology/Chemistry), University of Bremen, 330440, 28334 Bremen, Germany; 2 Centre for Environmental Research and Sustainable Technologies, University of Bremen, Bremen, Germany

**Keywords:** Astrocytes, Glucose, NADPH, Oxidative stress, pentose phosphate pathway

## Abstract

Glucose-6-phosphate dehydrogenase (G6PDH) catalyses the rate limiting first step of the oxidative part of the pentose phosphate pathway (PPP), which has a crucial function in providing NADPH for antioxidative defence and reductive biosyntheses. To explore the potential of the new G6PDH inhibitor G6PDi-1 to affect astrocytic metabolism, we investigated the consequences of an application of G6PDi-1 to cultured primary rat astrocytes. G6PDi-1 efficiently inhibited G6PDH activity in lysates of astrocyte cultures. Half-maximal inhibition was observed for 100 nM G6PDi-1, while presence of almost 10 µM of the frequently used G6PDH inhibitor dehydroepiandrosterone was needed to inhibit G6PDH in cell lysates by 50%. Application of G6PDi-1 in concentrations of up to 100 µM to astrocytes in culture for up to 6 h did not affect cell viability nor cellular glucose consumption, lactate production, basal glutathione (GSH) export or the high basal cellular ratio of GSH to glutathione disulfide (GSSG). In contrast, G6PDi-1 drastically affected astrocytic pathways that depend on the PPP-mediated supply of NADPH, such as the NAD(P)H quinone oxidoreductase (NQO1)-mediated WST1 reduction and the glutathione reductase-mediated regeneration of GSH from GSSG. These metabolic pathways were lowered by G6PDi-1 in a concentration-dependent manner in viable astrocytes with half-maximal effects observed for concentrations between 3 and 6 µM. The data presented demonstrate that G6PDi-1 efficiently inhibits the activity of astrocytic G6PDH and impairs specifically those metabolic processes that depend on the PPP-mediated regeneration of NADPH in cultured astrocytes.

## Introduction

Glucose-6-phosphat dehydrogenase (G6PDH) is the first and rate limiting enzyme of the oxidative part of the pentose phosphate pathway (PPP) [1, 2]. G6PDH oxidises glucose-6-phosphate (G6P) to 6-phosphogluconolactone which is hydrolysed to 6-phosphogluconate that subsequently serves as substrate of 6-phosphogluconate dehydrogenase (6PGDH). Both oxidoreductases of the oxidative part of the PPP use NADP^+^ as substrate and regenerate NADPH that is needed as electron donor for reductive biosyntheses and antioxidative processes in brain [3–5].

Among the different cell types of the brain, especially astrocytes have a prominent PPP [5]. In brain, astrocytes are highly important partners of neurons that provide metabolic substrates, regulate extracellular homeostasis and have important functions in detoxification processes and in the defence against oxidative stress [6–11]. A key component of the prominent antioxidative potential of astrocytes is the tripeptide glutathione (GSH) [9, 12–14]. During glutathione peroxidase-mediated reduction of peroxides or by chemical reactions with radicals cellular GSH is oxidised in astrocytes to glutathione disulfide (GSSG). The GSSG produced during such processes is efficiently reduced back to GSH by the cellular enzyme glutathione reductase (GR), which consumes NADPH as electron source [9, 14]. In order to maintain a high GSH to GSSG ratio, continuous regeneration of NADPH in the cytosol of astrocytes is required to supply GR with the electron donor needed. In unstressed conditions, the PPP contributes to less than 10% of the consumption of glucose in cultured astrocytes, but this contribution can be strongly enhanced to more than 50% by application of oxidative stress [15], demonstrating the importance and the high potential of PPP-derived NADPH regeneration for the antioxidative defence in astrocytes [3, 5, 15–17]. In addition, PPP-derived NADPH is an important electron donor for various other cytosolic enzymatic reactions in astrocytes [3], including the reactions that are catalysed by NADPH oxidase [12, 18], inducible nitric oxide synthase [19] and NAD(P)H:quinone oxidoreductase 1 (NQO1) [20, 21].

Pharmacological inhibition of G6PDH by dehydroepiandrosterone (DHEA) or 6-aminonicotinamide (6AN) has frequently been used to inhibit G6PDH and PPP activity in various cell types and models (for an overview see [1, 22]), including brain cells [16, 23–27]. However, the potential of such inhibitors to efficiently inhibit G6PDH activity has been disputed [28] and their specificity towards G6PDH is a concern since both inhibitors possess, especially during longer exposure times, well-known side effects as DHEA is a steroidal hormone [23] and 6AN is a known toxin [22, 25, 29].

Recently, G6PDi-1 has been reported as new and highly potent inhibitor of G6PDH and of PPP-dependent processes in human cells [28]. Little information is available so far on consequences of an exposure of brain cells to G6PDi-1. In cultured motor neurons, G6PDi-1 prevented the neuronal death induced by expression of the mutated proteins SOD1 or TDP43 in a model of amyotrophic lateral sclerosis, but did not affect the viability of neurons expressing the wildtype proteins [30]. We have recently used G6PDi-1 to elucidate whether PPP-derived NADPH is an electron source for the cytosolic NQO1 in cultured astrocytes and found that 10 µM G6PDi-1 inhibits 60% of the supply of electrons for NQO1-catalysed β-lapachone-mediated reduction of the water-soluble tetrazolium salt 1 (WST1) [21], suggesting that PPP-mediated NADPH is the main electron providing pathway for this process.

To investigate in more detail the potential of G6PDi-1 to inhibit G6PDH in astrocytes as well as its biocompatibility and potential to cause adverse consequences on the cell viability, the glucose and the glutathione metabolism of brain cells, we exposed cultured astrocytes to G6PDi-1 and investigated crucial metabolic processes. Here we report that G6PDi-1 efficiently and specifically inhibits astrocytic G6PDH without affecting other oxidoreductases, and impairs processes that depend on PPP-derived NADPH such as cellular NQO1-dependent WST1 reduction and cellular GSH regeneration from GSSG during oxidative stress in cultured astrocytes. In contrast, cell viability, glycolytic lactate production and the basal GSH metabolism are not acutely affected by the presence of G6PDi-1. These data demonstrate that G6PDi-1 is a suitable and specific tool to efficiently inhibit G6PDH and PPP-dependent processes in cultured brain cells.

## Materials and Methods

### Materials

Dulbecco’s modified Eagle’s medium (DMEM with 25 mM glucose) powder (12100061) and penicillin G/streptomycin sulfate (15140122) solution was from Thermo Fisher Scientific (Schwerte, Germany; RRID:SCR_008452). Fetal calf serum (FCS; F7524) was purchased from Sigma-Aldrich (Steinheim, Germany; RRID:SCR_008988). G6PDi-1 (31484) was obtained from Cayman Chemical (Tallinn, Estonia; RRID:SCR_008945), 6AN (A-0630) from Sigma-Aldrich (Steinheim, Germany; RRID:SCR_008988), DHEA (30770) from Fluka (Buchs, Switzerland), β-lapachone (ab141097) from Abcam (Berlin, Germany; RRID:SCR_012931), ES936 (sc-362737) from Santa Cruz Biotechnology (Heidelberg, Germany; RRID:SCR_008987) and WST-1 (W201) from Dojindo (Munich, Germany). All enzymes for the assays to quantify lactate, glucose and glutathione were purchased from Roche Diagnostics (Mannheim, Germany; RRID:SCR_001326). All other chemicals were obtained in the highest purity available from Merck (Darmstadt, Germany; RRID:SCR_001287), Fluka (Buchs, Switzerland), Sigma-Aldrich (Steinheim, Germany; RRID:SCR_008988), AppliChem (Darmstadt, Germany; RRID:SRC_005814) or Carl Roth (Karlsruhe, Germany; RRID:SCR_005711). Sterile cell culture material as well as non-sterile cups and microtiter plates were from Sarstedt (Nümbrecht, Germany).

### Astrocyte-Rich Cultures

Astrocyte-rich primary cultures were prepared from whole brains of new-born Wistar rats as previously described [31]. The rats were purchased from Charles River Laboratories (Sulzfeld, Germany; RRID:SCR_003792) and were treated according to the animal welfare acts of the State of Bremen, of Germany and of the European Union. The cells for the cultures were obtained by mechanical dissociation of the brain [31]. 300,000 viable cells were seeded in 1 mL of culture medium (90% DMEM containing 25 mM glucose, 10% FCS, 1 mM pyruvate, 18 U/mL penicillin G and 18 µg/mL streptomycin sulfate) into the wells of 24well plates or 3 × 10^6^ cells in 5 mL culture medium into 60 mm plates. The cultures were kept in culture medium at 37 °C with 10% CO_2_ in the humidified atmosphere of a cell incubator (Sanyo, Japan) to establish with the 44.6 mM bicarbonate of the DMEM a pH of 7.4. The cultures reached confluency within 2 weeks in culture. The high-glucose culture medium was renewed every 7 days and one day before an experiment. In this context it should be noted that culturing astrocyte cultures with glucose in hyperglycemic concentrations can modify metabolic processes in cultured astrocytes [32–34]. Experiments were performed on confluent astrocyte cultures of an age between 14 and 28 days. In this time range, no obvious age-dependent differences were observed for the specific parameters investigated in our study. Astrocyte-rich primary cultures contain more than 90% GFAP-positive astrocytes and only low amounts of contaminating other types of glial cells [31, 35].

### Determination of Enzyme Activities in Lysates of Cultured Astrocytes

The activity of cytosolic enzymes was determined in lysates of astrocyte cultures. Lysates were prepared as previously described [20]. Briefly, astrocytes grown on 60 mm dishes were washed once with 2 mL ice-cold phosphate-buffered saline (PBS; 10 mM KH_2_PO_4_/K_2_HPO_4_ pH 7.4 containing 150 mM NaCl), once with 2 mL ice-cold hypertonic potassium phosphate buffer (20 mM KH_2_PO_4_/K_2_HPO_4_ pH 6.5) and lysed in 1 mL ice-cold hypertonic potassium phosphate buffer for 30 min on ice. For the determination of NQO1 activity cells were lysed in 1 mL ice-cold hypertonic potassium phosphate buffer (20 mM KH_2_PO_4_/K_2_HPO_4_ pH 7.3) for 30 min on ice. The cells were scraped off the dish with the help of a cell scraper and subsequently transferred into a 1.5 mL reaction tube. After centrifugation of the lysate for 5 min at 12,000 g the supernatant was collected to determine the activity of cytosolic enzymes.

G6PDH activity was determined as described previously [36] using a modification of a previously reported method [37]. Briefly, the enzyme activity in 20 µL of the supernatant was determined in wells of a 96 well plate in a total volume of 200 µL containing final concentrations of 75 mM Tris/HCl buffer pH 7.5, 3.3 mM G6P, 0.2 mM NADP^+^, 6.3 mM MgCl_2_, 5 mM maleimide (to prevent additional NADPH production by inhibiting the 6PGDH activity in the lysate) and the given concentrations of inhibitors. Similarly, the activity of 6PGDH was determined for 20 µL of the supernatant in a total volume of 200 µL reaction volume containing final concentrations of 75 mM Tris/HCl buffer pH 7.5, 6.3 mM MgCl_2_, 2 mM 6-phosphogluconate, 0.2 mM NADP^+^, and 0 µM or 100 µM G6PDi-1. The increase in absorption at 340 nm was monitored at room temperature over 10 min in 15 s intervals and the enzyme activities were calculated by using the Lambert-Beer law and the molar extinction coefficient for NADPH of 6.2 mM^−1^ x cm^−1^ [31].

The activity of glutathione reductase (GR) in lysates of astrocyte cultures was determined as described previously [38]. In short, 120 µL of the supernatant were diluted in wells of a microtiter plate in a total reaction volume of 200 µL containing final concentrations 60 mM potassium phosphate buffer pH 7.0, 0.6 mM EDTA, 0.2 mM NADPH, 3.3 mM GSSG and no or 100 µM G6PDi-1. The decrease in absorption at 340 nm was monitored at 37 °C for 20 min in 10 s intervals and the enzyme activity was calculated by using the Lambert-Beer law and the molar extinction coefficient of NADPH of 6.2 mM^−1^ x cm^−1^ [31].

The NQO1 activity was determined by the reduction of the WST1 in the presence of the electron cycler menadione as described previously [20, 39]. For the assay, 10 µL of lysate were diluted in a total reaction volume of 200 µL in wells of a microtiter plate in glucose-free incubation buffer (IB; 145 mM NaCl, 20 mM HEPES, 5.4 mM KCl, 1.8 mM CaCl_2_, 1 mM MgCl_2_, 0.8 mM Na_2_HPO_4_, pH adjusted with NaOH to 7.4 at 37 °C) containing final concentrations of 70 µM WST1, 100 µM menadione and 5 mM NADH. Subsequently, the NQO1 activity was determined at room temperature from the linear increase in WST1 formazan absorbance at 450 nm by using the extinction coefficient of 35.2 mM^−1^x  cm^−1^ [39].

Specific enzyme activities were calculated by normalising the enzyme activities determined to the protein content of the lysate supernatant that had been quantified according to the Lowry method [40].

### Experimental Incubations of Cultured Astrocytes with G6PDi-1

To test for the effects of G6PDi-1 on basal metabolic parameters, confluent astrocyte cultures were washed twice with 1 mL pre-warmed (37 °C) glucose-containing incubation buffer (IB; 20 mM HEPES, 145 mM NaCl, 5.4 mM KCl, 1.8 mM CaCl_2_, 1 mM MgCl_2_, 0.8 mM Na_2_HPO_4_, 5 mM glucose, pH 7.4) and incubated at 37 °C with 200 µL IB that contained G6PDi-1 in the given concentrations of up to 100 µM in the humidified atmosphere (without CO_2_) of a cell incubator (Sanyo, Japan). After the given incubation periods, the media were harvested for determination of extracellular lactate dehydrogenase (LDH) activity and for the contents of extracellular glucose, lactate, total glutathione (GSx) and glutathione disulfide (GSSG). The cells were washed twice with 1 mL ice-cold (4 °C) PBS and lysed for quantification of GSx and GSSG.

To test for the effects of G6PDi-1 on the cell-dependent WST1 reduction, astrocyte cultures were washed twice with 1 mL pre-warmed (37 °C) glucose-free IB and preincubated at 37 °C with 300 µL glucose-free IB. After washing with 300 µL glucose-free IB, the cells were incubated in 300 µL glucose-free IB or 300 µL IB containing 5 mM glucose with 3 µM of the redox cycler β-lapachone, 400 µM WST1 and G6PDi-1 in the indicated concentrations for up to 60 min in the humidified atmosphere (without CO_2_) of a cell incubator. At the given time points the incubation media were collected and 50 µL media samples were diluted with water to a total volume of 200 µL in wells of a microtiter plate. The absorbance of the WST1 formazan generated was measured at 450 nm in a microtiter plate reader (Multiscan Sky, Thermo Fisher, Darmstadt, Germany) as previously reported [21, 39]. The specific WST1 formazan accumulation was calculated by normalisation of the extracellular WST1 formazan content to the initial protein content of the respective cultures.

To test for the effects of G6PDi-1 on the GSx and GSSG contents of astrocyte cultures during mild oxidative stress, the cells were washed twice with 1 mL pre-warmed (37 °C) glucose-containing (5 mM) IB and incubated at 37 °C with 300 µL glucose-containing IB that contained 5 µM β-lapachone and G6PDi-1 in the concentrations indicated (up to 100 µM) for up to 2 h in the humidified atmosphere (without CO_2_) of a cell incubator. After the given incubation periods, the media were harvested for determination of the extracellular LDH activity and the extracellular contents of GSx and GSSG. The cells were washed twice with 1 mL ice-cold (4 °C) PBS and lysed for quantification of the cellular contents of GSx and GSSG.

### Quantification of Extracellular Glucose and Lactate

The concentrations of glucose and lactate in the incubation media before and after a given incubation period of cultured astrocytes were determined by coupled enzymatic assays as described previously in detail [31]. Glucose consumption was calculated as difference between the initial glucose content of the incubation buffer applied to the cells and the glucose content determined after a given incubation period.

### Determination of Glutathione, Glutathione Disulfide, Protein and Cell Viability

The contents of cellular and extracellular total glutathione [GSx = amount of glutathione (GSH) plus twice the amount of GSSG] and GSSG were quantified by the modification in microtiter format of the colorimetric Tietze enzymatic cycling method as previously described in detail [31]. The initial protein content of the astrocyte cultures used for the given experiments was quantified by the Lowry method [40] using bovine serum albumin as standard protein. Potential impairment of cell viability of a given treatment was determined by testing for the release of the cytosolic enzyme LDH into the incubation medium by a photometric microtiter plate assay as described previously in detail [31].

### Data Analysis and Statistical Analysis

All data are presented are means ± standard deviations (SD) of values that are derived from experiments that have been performed on at least three independently prepared astrocyte cultures. The significance of differences between two data sets were analysed with a t-test and three or more data sets were analysed for significance by ANOVA followed by the Bonferroni *posthoc* test. The level of significance is indicated by asterisks or hashes (^#^ or *: p < 0.05, ^##^ or **: p < 0.01, ^###^ or ***: p < 0.001). p-values above 0.05 were considered as not significant.

## Results

### Inhibition of Astrocytic G6PDH Activity by G6PDi-1, DHEA or 6AN

**Fig. 1:**
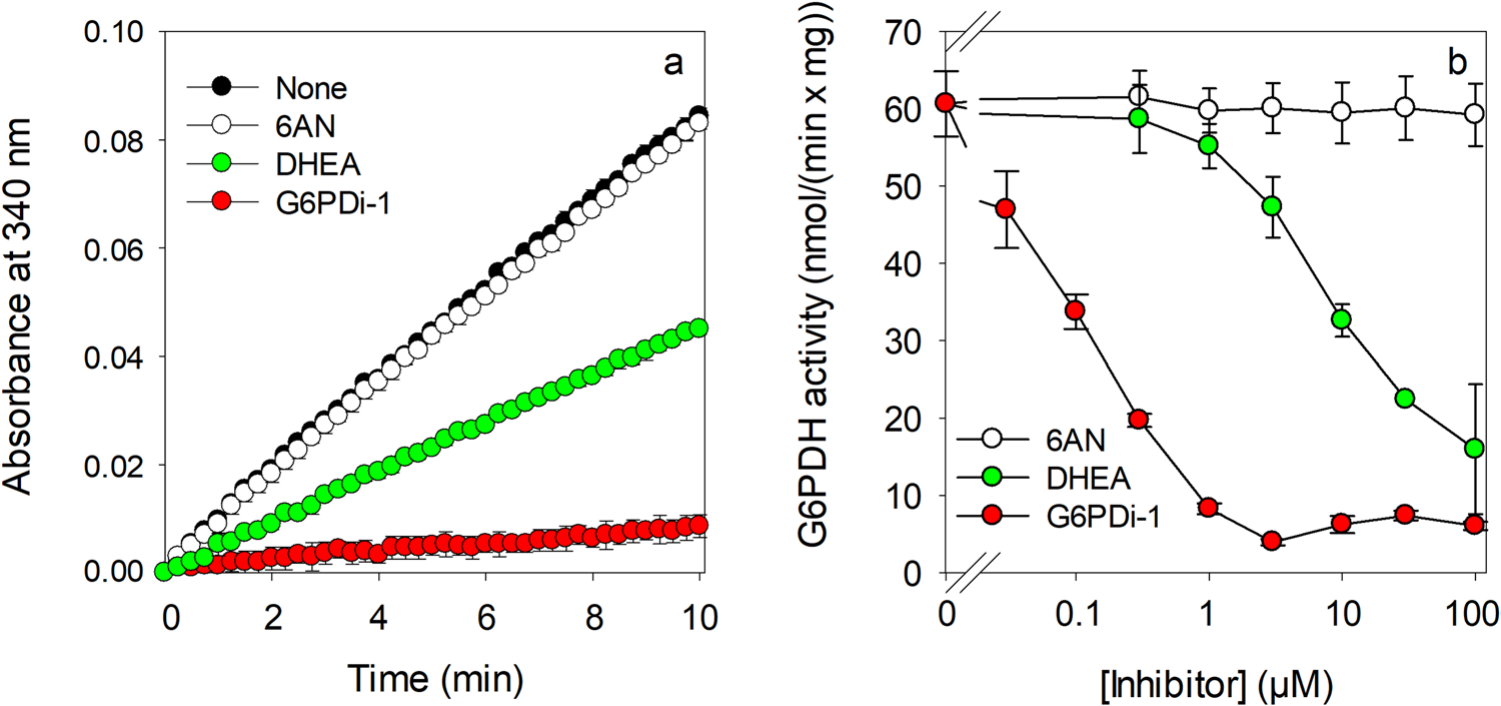
Effects of G6PDi-1, 6AN and DHEA on the activity of G6PDH in lysates of cultured astrocytes. The activity of the G6PDH was determined by the increase in NADPH absorbance at 340 nm in the absence or the presences of the indicated inhibitors. Panel **a** shows values from one representative experiment for 10 µM of the listed inhibitors. The linear increase in absorbance per minute was used to calculate the activity of G6PDH. Panel **b** shows the G6PDH activities calculated for the given concentrations of inhibitors. Half-maximal inhibition of G6PDH was found for 102 ± 3 nM of G6PDi-1 and for 9.2 ± 3.5 µM of DHEA. The protein content of the supernatant was 439 ± 19 µg per dish. The values in panel a are from one representative experiment that was reproduced twice with comparable results in independent experiments on lysates from other cultures. The data shown in panel b are means ± SD of values from independent experiments that had been performed on three independently prepared astrocyte cultures

To test for the potential of G6PDi-1 to inhibit astrocytic G6PDH, astrocyte primary cultures were lysed in hypotonic buffer and the soluble lysate supernatant was used for determination of G6PDH activity in the absence or the presence of G6PDi-1 (Fig. 1). The specific G6PDH activity in astrocytic lysate supernatants was determined to be around 60 nmol/(min x mg) (Fig. 1; Table 1). Presence of G6PDi-1 lowered the G6PDH-catalysed increase in NADPH absorption at 340 nm (Fig. 1a) in a concentration-dependent manner that corresponded to a severe decrease in the calculated G6PDH activity (Fig. 1b). Half-maximal inhibition was calculated for a G6PDi-1 concentration of 102 ± 3 nM, while G6PDH activity was inhibited by around 90% at inhibitor concentrations above 3 µM (Fig. 1b). In contrast, the presence of 6AN in concentrations of up to 100 µM did not lower G6PDH activity (Fig. 1a, b), while the presence of DHEA caused a concentration-dependent inhibition of astrocytic G6PDH activity with a half-maximal inhibition calculated for a concentration of 9.2 ± 3.5 µM (Fig. 1b).

**Table 1:** Test for modulation of enzyme activities by G6PDi-1. The specific activities of the listed enzymes were determined for lysates of primary astrocyte cultures in the absence (Control) or the presence of 100 µM G6PDi-1

Enzyme activity	Control	G6PDi-1
	(nmol/(min x mg))	(nmol/(min x mg))
Glutathione reductase	16.5 ± 2.9	16.8 ± 2.6
NQO1	453 ± 23	401 ± 2
G6PDH	60.6 ± 4.2	6.0 ± 0.6**
6PGDH	40.5 ± 1.6	34.3 ± 3.4*

The significance of differences (calculated by t-test) of the values obtained for incubations without and with G6PDi-1 are indicated by *p < 0.05 and **p < 0.01

Preliminary experiments on the G6P-dependence of the G6PDH activity revealed for the enzyme in lysates of astrocytes a hyperbolic Michaelis-Menten type correlation with a K_M_ value for G6P of around 130 µM that was not affected by the presence of G6PDi-1 in concentrations of up to 1 µM, while the calculated maximal activity was lowered by G6PDi-1 (data not shown). These results are consistent with the reported [28] non-competitive inhibition of G6PDH by the inhibitor G6PDi-1.

G6PDi-1 lowered the detectable activity of G6PDH in lysates of astrocytes almost completely in a concentration of 100 µM (Table 1). In contrast, this concentration of G6PDi-1 did not significantly lower the activity of NQO1 or GR determined for astrocyte lysates (Table 1). G6PDi-1 had also little effect on the activity of the 6PGDH, the second NADPH-producing enzyme in the PPP. 6PGDH activity was lowered by 100 µM G6PDi-1 by only 15 % compared to inhibitor-free controls (Table 1). These data demonstrate the high specificity of G6PDi-1 for G6PDH and that the effects observed for G6PDi-1-treated astrocytes are not caused by a potential unspecific inhibition of cellular GR or NQO1 by G6PDi-1.

### Test for Potential Consequences of an Exposure to G6PDi-1 on Glycolysis, Glutathione Content and Viability of Unstressed Astrocyte Cultures

**Fig. 2:**
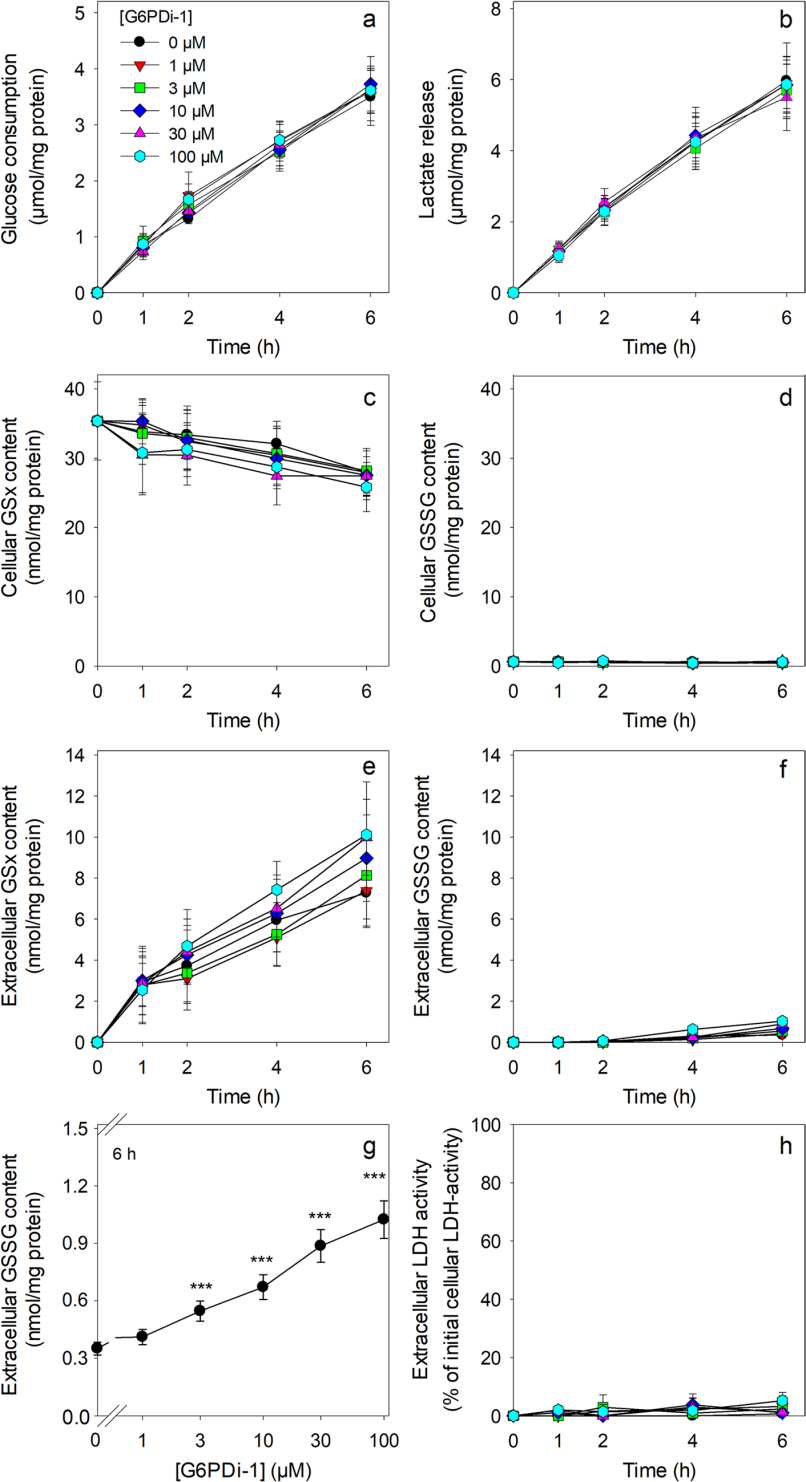
Test for potential effects of G6PDi-1 on the glucose consumption, the lactate release, the glutathione metabolism and the viability of cultured astrocytes. The cells were incubated without (0 µM) or with the given concentrations of G6PDi-1 in glucose-containing (5 mM) incubation buffer for up to 6 h. Glucose consumption (**a**), lactate release (**b**), cellular (**c**) and extracellular (**e**) GSx contents, cellular (**d**) and extracellular (**f**, **g**) GSSG contents as well as extracellular LDH activity (**h**) were measured for the indicated time points. The initial specific GSx content was 35.4 ± 5.6 nmol/mg protein and the specific GSSG content was 0.6 ± 0.1 nmol GSx/mg protein. The initial protein content of the cultures was 145 ± 15 µg per well. The data presented are means ± SD of values from experiments that had been performed on three independently prepared astrocyte cultures. The significance of differences (as calculated by ANOVA) compared with data for incubations without G6PDi-1 (0 µM) is indicated by ***p < 0.001

To test for potential adverse consequences of a treatment with G6PDi-1, cultured astrocytes were exposed to G6PDi-1 in different concentrations and the cell viability as well as several basal metabolic parameters were investigated (Fig. 2). Incubation of astrocytes with up to 100 µM G6PDi-1 for up to 6 h did not cause any alteration in cellular glucose consumption (Fig. 2a) or in lactate release (Fig. 2b) nor was the cell viability compromised by these treatments (Fig. 2h). In addition, the basal slow disappearance of cellular GSx during the incubation (Fig. 2c), the low cellular GSSG content (Fig. 2d) and the extracellular accumulation of GSx (Fig. 2e) were not significantly altered compared to control cells (absence of G6PDi-1) during an incubation of astrocytes for up to 6 h with G6PDi-1 in concentrations of up to 100 µM. These results revealed that exposure of astrocytes to G6PDi-1 in concentrations of up to 100 µM does, under the conditions used, not affect cell viability, glycolytic lactate production or the basal GSH metabolism of cultured astrocytes. Only during an extended exposure of astrocytes with high concentrations of G6PDi-1 a significant, but marginal increase in the extracellular content of GSSG from around 0.3 nmol/mg (controls) to around 1 nmol/mg (100 µM G6PDi-1) was detected (Fig. 2f, g) that corresponded to less than 10% of the total extracellular GSx levels (Fig. 2e).

### WST1 Reduction by Astrocytes in the Absence or the Presence of G6PDi-1

**Fig. 3:**
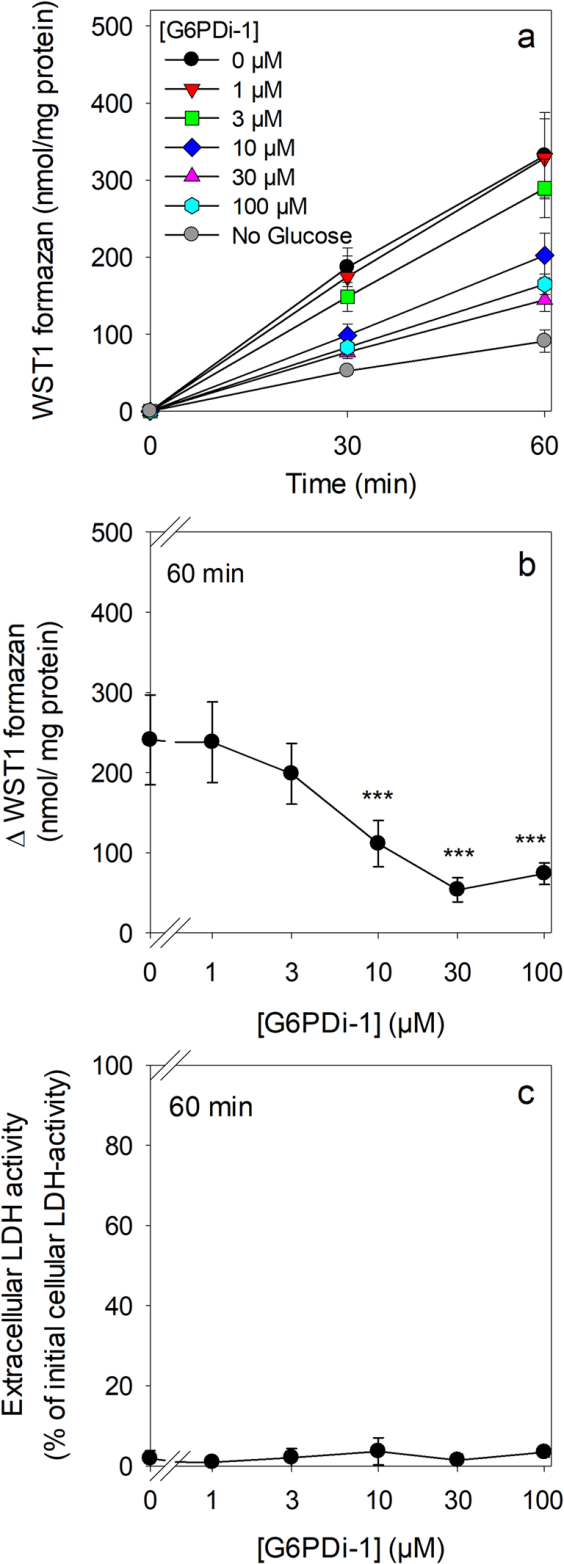
Time- and concentration-dependent effects of G6PDi-1 on NQO1-catalysed and β-lapachone-mediated WST1 reduction by cultured astrocytes. The cells were preincubated for 30 min in the absence of glucose and subsequently incubated with 5 mM glucose, 400 µM WST1 and 3 µM β-lapachone in the absence (0 µM) or the presence of the indicated concentrations of G6PDi-1 for up to 60 min. The extracellular WST1 formazan content (a, b) and the extracellular LDH activity (c) were determined for the indicated time points. The initial protein content of the cultures was 154 ± 16 µg per well. The data presented are means ± SD of values from experiments that had been performed on three independently prepared astrocyte cultures. In panel b, the significance of differences (as calculated by ANOVA) compared with data for incubations without G6PDi-1 (0 µM) is indicated by ***p < 0.001

NQO1-dependent β-lapachone-mediated reduction of extracellular WST1 requires intracellular NADPH as electron source in a process that has been reported to be at least partially inhibited by the presence of 10 µM G6PDi-1 [21]. To test for the concentration-dependent potential of G6PDi-1 to affect astrocytic WST1 reduction, astrocytes were incubated with 3 µM β-lapachone and 400 µM WST1 in the absence or the presence of G6PDi-1 in concentrations of up to 100 µM. In the absence of the inhibitor, an almost linear extracellular accumulation of WST1 formazan was observed that lead to the extracellular formation of around 330 nmol/mg WST1 formazan within 60 min of incubation (Fig. 3a). In the absence of glucose, a much slower increase in extracellular WST1 accumulation was observed, yielding around 90 nmol/mg WST1 formazan within 60 min of incubation (Fig. 3a). The glucose-dependent cellular WST1 reduction (difference between the values quantified after incubation in the presence and in the absence of glucose) was strongly lowered by G6PDi-1 in a concentration-dependent manner (Fig. 3a) with half-maximal inhibition observed at a concentration of around 6 µM (Figs. 3b and 5). Application of G6PDi-1 in a concentration of 100 µM lowered the total cellular WST1 reduction determined after 60 min incubation by 50% (Fig. 3a) and the glucose-dependent WST1 reduction by 69 % (Fig. 3b).

The contribution of the enzyme NQO1 in the observed astrocytic WST1 reduction was confirmed by the application of the NQO1 inhibitor ES936 [21, 41]. This inhibitor lowered in a concentration of 3 µM both the total WST1 reduction as well as the residual WST1 reduction observed in the presence of 100 µM G6PDi-1 by around 90% (Table 2). None of the conditions applied caused any significant loss in cell viability as demonstrated by the absence of any significant increase in extracellular LDH activity compared to the control incubations (Fig. 3c; Table 2).

**Table 2:** Effects of G6PDi-1 and the NQO1 inhibitor ES936 on the β-lapachone-mediated WST1 reduction by astrocytes

	LDH	WST1 formazan content
Condition	(%)	(nmol/mg protein)
No inhibitor	2 ± 2	332 ± 56
100 µM G6PDi-1	3 ± 1	165 ± 13***
3 µM ES936	3 ± 3	37 ± 1***
100 µM G6PDi-1 + 3 µM ES936	2 ± 1	17 ± 2***

The cells were preincubated for 30 min in the absence of glucose and subsequently incubated for 60 min with 5 mM glucose, 400 µM WST1 and 3 µM β-lapachone in the absence (0 µM) or the presence of the indicated inhibitors. After 60 min incubation, the extracellular WST1 formazan content and the extracellular LDH activity (given as percent of the initial cellular LDH activity) were measured. The data listed for the control (No inhibitor) and for the 100 µM G6PDi-1 treatment are taken from Fig. 3. The initial protein content of the cultures was 154 ± 16 µg per well. The data presented are means ± SD of values from experiments that had been performed on three independently prepared astrocyte cultures. The significance of differences (as calculated by ANOVA) compared with data for incubations without inhibitor is indicated by ***p < 0.001

**Table 3:** Effects of G6PDi-1 and ES936 on the β-lapachone-induced oxidative stress in astrocytes

	LDH	GSSG content (nmol GSx/ mg protein)		GSx content (nmol/mg protein)		Glucose consumption (µmol/mg protein)	Lactate release (µmol/mg protein)	Lactate release / Glucose consumption
Condition	(%)	Cellular	Extracellular	Cellular	Extracellular	Extracellular	Extracellular	Extracellular
	(120 min)	(10 min)	(120 min)	(120 min)	(120 min)	(120 min)	(120 min)	(120 min)
No inhibitor	9 ± 7	7 ± 5	8 ± 10	31 ± 5	15 ± 11	1.75 ± 0.32	2.78 ± 0.80	1.6 ± 0.3
100 µM G6PDi-1	7 ± 4	24 ± 6**	27 ± 8**	9 ± 1**	37 ± 6**	1.55 ± 0.09	2.07 ± 0.09	1.3 ± 0.1
30 µM ES936	12 ± 7	1 ± 1	2 ± 2	36 ± 10	10 ± 6	1.61 ± 0.23	2.86 ± 0.99	1.7 ± 0.4
G6PDi-1 + ES936	5 ± 5	1 ± 1	3 ± 2	37 ± 9	14 ± 1	1.88 ± 0.15	2.50 ± 0.33	1.3 ± 0.3

The cells were incubated without (No inhibitor) or with G6PDi-1 (100 µM) and/or the NOQ1 inhibitor ES936 (30 µM) in the presence of 5 µM β-lapachone in glucose-containing (5 mM) incubation buffer for up to 2 h. Cellular and extracellular GSx and GSSG contents, extracellular LDH activity (given as percent of the initial cellular LDH activity) as well as glucose consumption, lactate release and the ratio of lactate release to glucose consumption were measured for the indicated time points. The initial specific GSx content of the cultures was 45.5 ± 9.0 nmol/mg protein and the specific GSSG content was 0.1 ± 0.1 nmol GSx/mg protein. The initial protein content of the cultures was 167 ± 34 µg per well. The data presented are means ± SD of values from experiments that had been performed on three independently prepared astrocyte cultures. The significance of differences (as calculated by ANOVA) compared with data for incubations without inhibitor (No inhibitor) is indicated by **p < 0.01

### Effects of G6PDi-1 on the β-lapachone-induced GSSG accumulation in cultured astrocytes

**Fig. 4:**
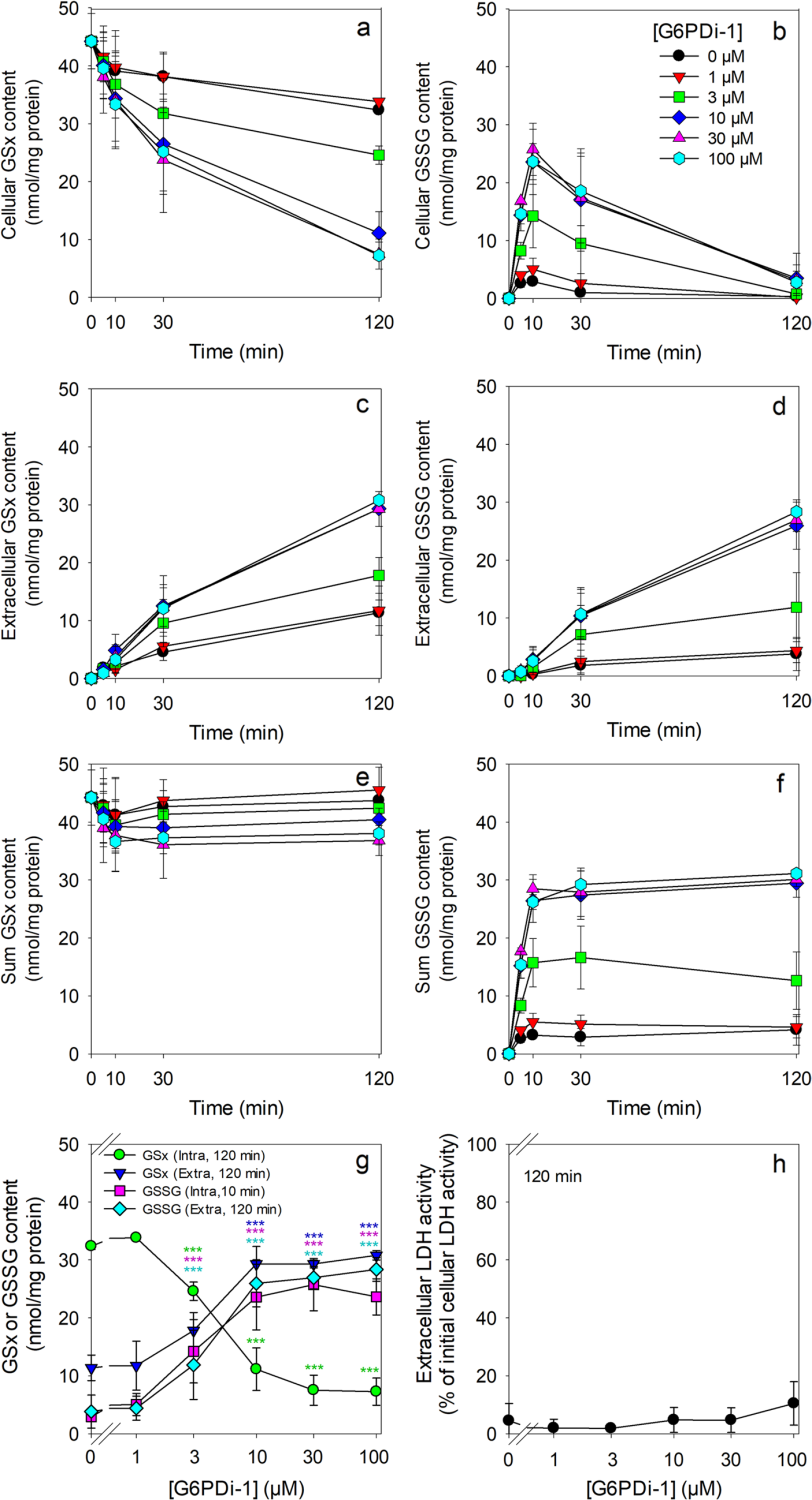
Enhancement of β-lapachone-induced oxidative stress by G6PDi-1 in cultured astrocytes. The cells were incubated without (0 µM) or with the indicated concentrations of G6PDi-1 in the presence of 5 µM β-lapachone in glucose-containing (5 mM) incubation buffer for up to 2 h. Cellular (**a**) and extracellular (**c**) GSx contents, cellular (**b**) and extracellular (**d**) GSSG contents and extracellular LDH activity (**h**) were measured for the indicated time points. Panels e and f show the sum of intracellular plus extracellular GSx contents (**e**) and the sum of intracellular plus extracellular GSSG contents (**f**). The concentration-dependent effects of G6PDi-1 on the intra- und extracellular GSx and GSSG contents for the indicated time points is given in panel g. The initial specific GSx content of the cultures was 44.2 ± 4.8 nmol/mg protein and the initial specific GSSG content was 0.1 ± 0.2 nmol GSx/mg protein. The initial protein content of the cultures was 167 ± 35 µg per well. The data presented are means ± SD of values from experiments that had been performed on three independently prepared astrocyte cultures. In panel g, the significance of differences (as calculated by ANOVA) compared with data for incubations without inhibitor (0 µM) is indicated by ***p < 0.001 in the colours of the symbols indicating the measured parameters

The redox cycler β-lapachone has been reported to induce oxidative stress in cultured astrocytes in concentrations above 3 µM as demonstrated by a rapid cellular accumulation of GSSG that is followed by a subsequent extracellular accumulation of GSSG [42]. As the PPP is involved in the regeneration of the NADPH that is needed for GSSG reduction in astrocytes [5, 17], we tested for the potential of G6PDi-1 to impair cellular GSSG reduction and thereby to alter the cellular ratio of GSH to GSSG. Cultured astrocytes were exposed to the low concentration of 5 µM β-lapachone (in the absence of WST1) to induce a mild oxidative stress within the cells [42]. Indeed, application of 5 µM β-lapachone caused a moderate and transient increase in cellular GSSG content within 10 min from an initial value of 0.1 ± 0.2 nmol/mg of untreated cells to 2.9 ± 0.2 nmol/mg which represented 7% of the cellular GSx content of the treated cells (Fig. 4a, b). For this inhibitor-free condition (black circles), a slow loss in cellular GSx was observed during 120 min of incubation (Fig. 4a) as well as a slow extracellular accumulation of GSx (Fig. 4c) of which 34% represented GSSG (Fig. 4d). The total GSx content (cellular plus extracellular GSx content) of astrocytes that had been exposed to 5 µM β-lapachone remained constant (Fig. 4e) and the total GSSG content of the cultures accounted during the entire incubation only to around 10% of the total GSx content of the culture (Fig. 4f). The cells remained viable under these conditions as indicated by the low extracellular LDH activity that was detected after 120 min for this condition (Fig. 4h).

**Fig. 5:**
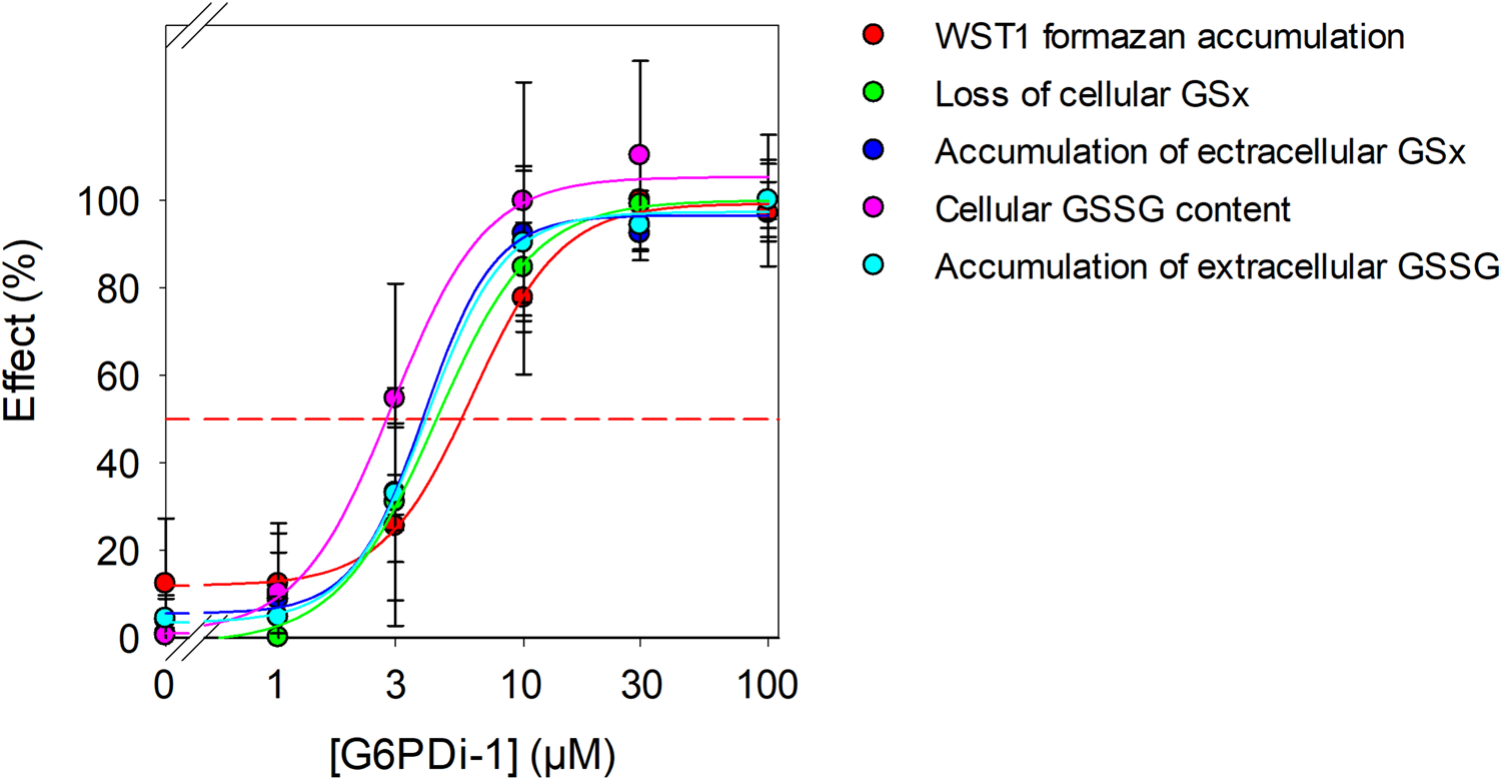
Comparison of the concentration-dependent effects of G6PDi-1 on the investigated parameters of cultured astrocytes. The specific values of cellular parameters that were found to be strongly affected in a concentration-dependent manner by the presence of G6PDi-1 (Table 4) were used to calculate the percental effect concentrations. For the calculations of the concentration-dependent effects of the parameters investigated, the values determined for the incubation with 100 µM G6PDi-1 were set as 100% and the values for the respective controls (absence of G6PDi-1) as 0%. From the curves obtained, the EC50 value were calculated and are given in Table 4. The colours of the symbols are indicating the investigated metabolic parameter (for more details see Table 4). The red dashed line is showing the 50% effects

Co-application of G6PDi-1 with 5 µM β-lapachone did also not compromise the viability of astrocytes (Fig. 4h), but the presence of G6PDi-1 accelerated the cellular loss of GSx in a concentration-dependent manner (Fig. 4a) and the extracellular GSx accumulation (Fig. 4c). The transient increase in cellular GSSG within 10 min of incubation was found to be much stronger in G6PDi-1-treated astrocytes compared to the respective controls. For example, the cellular GSSG levels of cells that had been exposed to 30 µM G6PDi-1 increased to around 26 nmol/mg, representing 76% of the cellular GSx content (Fig. 4b). Half-maximal effects of G6PDi-1 on the cellular accumulation of GSSG were found for inhibitor concentrations of around 3 µM (Figs. 4g, 5). The accelerated decline in cellular GSSG levels during longer incubation periods in the presence of G6PDi-1 (Fig. 4b) was accompanied by an increase in extracellular GSSG content (Fig. 4d), while the total amounts of GSx in the culture remained unaffected by the presence of G6PDi-1 (Fig. 4e). Additionally, the sum of cellular plus extracellular GSSG remained almost constant during incubation of the cells for more than 10 min and accounted for around 40% of the initial GSx content for cells that had been treated with 3 µM G6PDi-1 and to around 80% for cells exposed to higher concentrations of the inhibitor (Fig. 4f). The concentrations of G6PDi-1 that caused half-maximal effects on the loss in cellular GSx content and on the extracellular GSSG accumulation within 120 min of incubation was calculated to be around 4 µM (Figs. 4g, 5).

**Table 4:** Half-maximal effects of G6PDi-1 on astrocytic metabolic processes that involve G6PDH-dependent NADPH regeneration

	Original data	Incubation time	EC50
Test for	Figures	(min)	(µM)
WST1 reduction	3b	60	6.3 ± 2.2
Cellular loss of GSx	4a	120	4.2 ± 0.8
Extracellular GSx accumulation	4c	120	4.7 ± 2.0
Cellular GSSG accumulation	4b	10	3.2 ± 1.1
Extracellular GSSG accumulation	4d	120	4.4 ± 1.7

The concentrations of G6PDi-1 that inhibit half of the metabolic parameters investigated were calculated from the sigmoidal curves shown in Fig. 5

All effects of G6PDi-1 on the cellular and extracellular GSx and GSSG contents of β-lapachone-treated astrocytes were completely prevented by the co-application of the NQO1 inhibitor ES936 , demonstrating that the effects observed depend on NQO1-catalysed β-lapachone-mediated oxidative stress (Table 3). Determination of the glucose consumption, lactate release and of the ratio of lactate release to glucose consumption for the 120 min incubation time did not reveal any significant differences between the conditions investigated (Table 3).

### Comparison of the Concentration-Dependent Effects of G6PDi-1 on the Parameters Investigated for Cultured Astrocytes

To compare the strong concentration-dependent effects observed for treatments of astrocytes with G6PDi-1, the values determined were normalized to the respective maximal effect values (set as 100%) that was observed for a treatment with 100 µM G6PDi-1 (Fig. 5). The data summarized in Fig. 5 demonstrate that G6PDi-1 strongly affected astrocytic WST1 reduction as well as cellular GSSG accumulation and subsequent GSSG export from astrocytes with half-maximal effects observed for inhibitor concentrations between 3 and 6 µM (Table 4). Maximal effects of G6PDi-1 were already observed for 30 µM as the presence of 100 µM G6PDi-1 did not significantly further increase the effects observed (Fig. 5).

## Discussion

G6PDi-1 has recently been reported and characterized as efficient inhibitor of purified human G6PDH and of G6PDH-dependent processes in peripheral cells [28]. Here we have characterized this inhibitor for its potential to inactivate G6PDH in cultured primary rat astrocytes. G6PDi-1 inhibited the G6PDH activity in astrocyte lysates efficiently with half-maximal inhibition observed at an inhibitor concentration of around 100 nM. The inhibition by G6PDi-1 of G6PDH from rat astrocytes was found to be non-competitive, consistent with data reported for the human enzyme [28]. The concentration of G6PDi-1 needed to achieve half-maximal inhibition of the astrocytic G6PDH (100 nM) was quite similar to that reported for the purified human enzyme (70 nM) [28]. Species differences of the G6PDH investigated but also the presence of components in the astrocyte lysate that may bind part of the applied inhibitor and renders it unavailable for G6PDH inhibition could contribute to the slightly lower apparent inhibitory potential of G6PDi-1 for G6PDH in astrocyte lysates compared to that reported for the purified human enzyme.

Comparison of the inhibitory potential of G6PDi-1 on astrocytic G6PDH with that of DHEA and 6AN revealed that DHEA had to be present in 100fold higher concentration than G6PDi-1 to inhibit G6PDH by 50%, while 6AN even in a concentration of 100 µM did not show any G6PDH inhibition. The lower power of DHEA to inhibit G6PDH, compared to G6PDi-1, confirms literature data [28]. Also the inability of 6AN to directly inactivate G6PDH in lysates was expected, as 6AN is considered to act as inhibitor only after incorporation by cellular metabolism into the 6AN-analoge of NADP^+^ [22]. Thus, a treatment of astrocytes with 6AN will need some incubation time for cellular activation before the inhibitory potential of a 6AN exposure on G6PDH and PPP function will take place.

Investigation of potential inhibitory effects of G6PDi-1 on other enzymes that either produce NADPH (6PGDH) or consume NADPH (GR and NQO1) revealed that G6PDi-1, even if applied in a high concentration of 100 µM, hardly affected the other enzymes. Thus, G6PDi-1 appears to be a highly potent and specific inhibitor of astrocytic G6PDH and was therefore tested in further experiments for its potential to affect PPP-dependent processes in cultured astrocytes.

Application of G6PDi-1 to astrocyte cultures revealed that G6PDi-1 does not compromise cell viability, but strongly affects cellular processes that depend on PPP-mediated NADPH regeneration already in low micromolar concentrations, such as the NQO1-dependent WST1 reduction and the GR-dependent GSSG reduction. The presence of G6PDi-1 impaired the glucose-dependent astrocytic β-lapachone-mediated WST1 reduction in a concentration-dependent manner, a process that uses NADPH provided via the PPP as electron donor for the enzyme NQO1 to reduce the redox cycler β-lapachone [21]. In contrast, the presence of G6PDi-1 did not affect glycolytic glucose consumption or lactate production, suggesting that G6PDi-1 does not interfere with astrocytic glycolysis. In this respect G6PDi-1 differs strongly to inhibitors of glyceraldehyde-3-phosphate dehydrogenase that lower glycolytic lactate production [36, 43] or to inhibitors of mitochondrial respiration that stimulate glycolytic lactate production by astrocytes [44, 45]. The strong inhibitory effect of G6PDi-1 on astrocytic WST1 reduction and the absence of any effect of this inhibitor on glycolysis, supports the view [21] that PPP-derived NADPH is the main source of electrons for cytosolic NQO1 in cultured astrocytes.

PPP-derived NADPH is also required for the GSH regeneration from GSSG by GR in astrocytes [3, 38] and thereby helps to maintain the high ratio of GSH to GSSG normally found in unstressed astrocytes [21, 42, 46]. However, during oxidative stress cellular GSSG accumulates as consequence of an insufficient GSSG reduction capacity that can not match the speed of GSSG formation[42, 46]. Only a small transient increase in cellular GSSG was found within 10 min after application of 5 µM β-lapachone (in the absence of WST1) to astrocytes. This indicates that under such conditions a mild oxidative stress occurs in the cells as consequence of a slightly disturbed balance between prooxidative (NQO1-catalysed β-lapachone reduction) and subsequent superoxide production [42] and antioxidative (GR-catalysed NADPH-dependent GSSG reduction) processes [3]. However, this balance is dramatically affected by the presence of G6PDi-1 as demonstrated by the strong increase in cellular GSSG levels that was observed in β-lapachone-treated astrocytes in the presence of the inhibitor which is likely to be the direct consequence of the strong impairment of the NADPH regeneration via the PPP that is needed to provide the NADPH for GR-catalysed GSSG reduction [42, 46]. A potential inhibition of cellular GR or NQO1 by G6PDi-1 could also explain the observed GSSG accumulation, but appears to be highly unlikely as the presence of 100 µM G6PDi-1 did not affect the activity of astrocytic GR or NQO1.

During extended exposure of astrocytes to β-lapachone in the presence of G6PDi-1 cellular GSH and GSSG disappeared and GSSG accumulated extracellularly. These processes are caused by the strongly elevated cellular GSSG level that facilitates the export of GSSG via the multidrug resistance protein (Mrp) 1 [47–49].

Application of G6PDi-1 to unstressed cultured astrocytes revealed that presence of the inhibitor in concentrations of up to 100 µM does not affect the basal glycolytic lactate production by astrocytes nor the cell viability for incubations of up to 6 h, demonstrating the low toxic potential of G6PDi-1 for the conditions investigated. Such a low toxic potential was expected, as the PPP contributes in unstressed astrocytes less than 10% [15] to the glucose-6-phosphate consumption. Under such conditions, other cytosolic NADPH generating astrocytic enzymes, such as NADP^+^-dependent isocitrate dehydrogenase [50] and malic enzyme [51] could compensate for the impaired NADPH regeneration by the PPP in G6PDi-1-treated unstressed astrocytes. The application of G6PDi-1 to cultured astrocytes appears to not cause any obvious oxidative stress. At least no detectable increase in cellular GSSG contents was observed, supporting the view that unstressed astrocytes can metabolically compensate for an inhibition of the oxidative part of the PPP.

In conclusion, G6PDi-1 in concentrations of up to 100 µM does not compromise cell viability or basal GSH metabolism of cultured rat astrocytes nor glycolytic lactate production, but efficiently impairs already in low micromolar concentrations metabolic processes that depend on the regeneration of cytosolic NADPH by G6PDH and the PPP. G6PDi-1 appears to be a highly potent and rather specific inhibitor of G6PDH that should be considered as suitable tool for studies investigating potential involvement of NADPH regeneration by G6PDH and/or functions of the oxidative part of the PPP in brain cells. Application of G6PDi-1 is likely to be a good alternative to other established PPP inhibitors such as DHEA or 6AN, that have previously been used to target G6PDH or the PPP activity in brain cells [16, 23–27].
